# Unequal Efficacy of Different Infarct Location in Predicting Futile Recanalization of Patients With Acute Ischemic Stroke

**DOI:** 10.3389/fneur.2022.928773

**Published:** 2022-08-26

**Authors:** Zhao-shuo Li, Hai-long Zhong, Teng-fei Zhou, Ying-kun He, Qiang Li, Zi-liang Wang, Liang-fu Zhu, Chang-ming Wen, Jian-feng Han, Tian-xiao Li

**Affiliations:** ^1^Department of Cerebrovascular Disease, Zhengzhou University People's Hospital, Zhengzhou, China; ^2^Department of Neurology, Nanyang Central Hospital, Nanyang, China; ^3^Department of Neurology, Xi'an Jiaotong University Medical College First Affiliated Hospital, Xi'an, China

**Keywords:** stroke, endovascular treatment, Alberta Stroke Program Early Computed Tomography score, futile recanalization, predictive factor

## Abstract

**Objectives:**

Endovascular thrombectomy (EVT) is a standard treatment for acute ischemic stroke (AIS) caused by large vessel occlusion, while futile recanalization is the main factor influencing the prognosis. The present study aimed to investigate the efficacy of different infarct sites in predicting futile recanalization of patients with AIS.

**Methods:**

Data were obtained from two multicenter, prospective, randomized, and controlled trials, which were concurrently conducted in China. Cases achieving a successful recanalization and with complete data of preoperative Alberta Stroke Program Early CT score (ASPECTS) and 90-day follow-up were included. The ASPECTS subregions were used to mark different infarct locations in the two cerebral hemispheres. First, the distribution of each ASPECTS subregion in the left and right hemispheres and the whole brain was analyzed, respectively. Then, the regions associated with futile recanalization were initially assessed by a univariate model. Afterward, a multivariate logistic regression model was used to identify the efficacy of different infarct sites in predicting futile recanalization.

**Results:**

A total of 336 patients were included in this study with a median age of 65 years (IQR: 51–74), of whom 210 (62.50%) patients were male, and 189 (56.25%) met the definition of futile recanalization. The correlation between each ASPECTS subregion and poor outcome was different when it was restricted to a specific cerebral hemisphere. Moreover, in the left hemisphere, the internal capsule region (OR: 1.42, 95%CI: 1.13–1.95, *P* = 0.03) and the M3 region (OR: 2.26, 95%CI: 1.36–3.52, *P* = 0.001), and in the right hemisphere, M6 region (OR: 2.24, 95%CI: 1.32–3.36, *P* = 0.001) showed significantly higher efficacy in predicting futile recanalization.

**Conclusion:**

The efficacy of different infarct locations in predicting futile recanalization is different. Different preoperative patterns of the high-efficiency regions in the infarction core or penumbra can guide the thrombectomy decision-making.

## Introduction

In 2015, multiple randomized controlled trials (RCTs) demonstrated the superiority of endovascular therapy (EVT) over medical management alone for the treatment of acute ischemic stroke (AIS) due to large vessel occlusion (LVO) (1). With continuously updating of the thrombectomy devices and optimization of the techniques, the recanalization rate has noticeably increased, while more than 50% of patients who achieved successful recanalization [extended Thrombolysis in Cerebral Infarction Classification (eTICI) grade ≥ 2b] still could not have a good functional outcome [modified Rankin scale (mRS) score ≤ 2] at 90-day follow-up, and these patients were diagnosed as futile recanalization (2, 3).

Previous studies have explored several predictors of futile recanalization (4), of which preoperative infarct core has noticeably attracted clinicians' attention. However, the relevant studies mainly concentrated on the prediction of its volume (1, 5), the prognosis of patients with the same infarct volume may remarkably differ, suggesting that other parameters may also significantly influence the outcomes (6). For instance, in terms of the infarct topography, different regions and different brain hemisphere are likely to have different efficacy affecting prognosis (7, 8). If this hypothesis is confirmed, regions with high weight are located preoperatively in the infarct core or penumbra may be an important factor in making treatment decisions. Previous relevant studies have not presented a consistent conclusion yet (6–8), although the majority of them used the Alberta Stroke Program Early CT score (ASPECTS) subregions to mark infarct location. Therefore, in the present study, we retrospectively combined data from two multicenter, prospective RCTs with large sample sizes (Skyflow study, ChiCTR1800015166; Jrecan study, ChiCTR-TOC-17013822), which were concurrently conducted in China (9), to explore the efficacy of different infarct sites in predicting futile recanalization of patients with AIS.

## Methods

### Subjects

Both RCTs separately evaluated the efficacy of two new devices (Skyflow and JRecan) for the treatment of AIS due to LVO in the anterior circulation, and the RCTs were almost concurrently completed from 2018 to 2019 but in the different recruitment centers (Supplementary Table 1). The results of both RCTs have passed the inspection of the China Food and Drug Administration (FDA) and been published in China, and two new devices have been certified and applied to the daily clinic (10). The same inclusion criteria of both RCTs: (1) patients aged ≥18 years old; (2) time from symptom onset to arterial puncture was ≤8 h; (3) occlusion site located in the intracranial internal carotid, or M1/M2 segment of the middle cerebral artery (MCA) confirmed by computed tomography angiography (CTA) or digital subtraction angiography (DSA); (4) the National Institutes of Health Stroke Scale (NIHSS) score used to assess the stroke severity ≥6; (5) preoperative ASPECTS ≥6. The same exclusion criteria: (1) preoperative modified Rankin scale (mRS) score ≥2; (2) acute infarct involving in bilateral or multiple vascular areas; (3) pregnant or breast-feeding females; (4) patients involved in other drugs or equipment trials before randomization; (5) patients not receiving endovascular treatment after randomization. In both cohorts, the successful recanalization was defined as eTICI grade≥2b (11), and the good functional outcomes were defined as mRS score ≤2 at 90-day follow-up.

In the present study, data were re-screened and imported into a new database for analysis according to the following exclusion criteria. (1) According to the definition of futile recanalization (12, 13), patients who failed in recanalization or those without complete follow-up data were excluded. (2) As different preoperative infarct sites were marked using the ASPECTS subregions, cases with ASPECTS 10 were excluded. Referring to the Strengthening the Reporting of Observational Studies in Epidemiology (STROBE) statement (14), study subjects were divided into the futile recanalization (FR) group and the effective recanalization (ER) group.

### Clinical and Follow-Up Assessments

Baseline data of enrolled patients, namely, age, gender, stroke-associated risk factors (hypertension, diabetes, hyperlipidemia, and atrial fibrillation), and history of anticoagulation and antiplatelet therapy were collected. The NIHSS scores were assessed by two senior neurologists who were blinded to the other data for each case. Collateral grades were assessed with the American Society of Interventional and Therapeutic Neuroradiology/Society of Interventional Radiology (ASIT*N*/SIR) scale on angiography by two senior radiologists blinded to the clinical data (15). For cases with cerebral perfusion images, the infarct core volume of these patients was calculated using RAPID software. Cases who underwent intravenous thrombolysis followed by EVT were considered as those who received bridging therapy. The time of onset to admission, admission to imaging examination, and onset to recanalization was recorded. Changes in NIHSS scores at 24 h postoperative compared with preoperative were calculated to assess the short-term efficacy. Perioperative hemorrhage complications were classified according to the Heidelberg classification (16). The symptomatic intracerebral hemorrhage (sICH) was defined as radiologically proven intracranial hemorrhage accompanied by a clinical deterioration (≥4 points in the NIHSS) that could not be attributed to other causes.

### ASPECTS Subregions Assessments

Preoperative ASPECTS and infarcted subregions were recorded for all the included cases. When NCCT was used to calculate ASPECTS, in consideration of the poor consistency of manual measurement of NCCT-ASPECTS, automated measurement was alternatively conducted using RAPID software (17, 18). In contrast, because of the better consistency in identifying abnormal diffusion-weighted imaging (DWI) signals, the DWI-ASPECTS was rated by two senior radiologists blinded to the clinical data for each case (19).

For an ASPECTS region of early ischemic change, 1 point was subtracted, which was defined as the presence of hypoattenuation and/or loss of gray matter–white matter differentiation, with or without cortical swelling. The pre-existing infarct regions were defined as very obvious areas of hypoattenuation, which was based on the concept that very definite hypoattenuation does not occur within 3 h of stroke onset, and did not score (20). The abnormal signal volume in the ASPECTS subregions should be >1 ml before scoring, and no score was given for punctate infarction. For the infarct-involved boundary areas, the area most impacted was rated a point, whereas the other areas did not score.

As there was no patient with infarction core simultaneously affecting bilateral cerebral hemispheres in the present study, a total of 20 regions (10 ASPECTS subregions for each hemisphere) were considered. Each subregion was delineated as a region of interest (ROI). Three regions at the level of the basal ganglia were classified as deep perforator regions: caudate (C), lenticular nucleus (L), and internal capsule (IC). Insula (I) and the other six regions (M1–M6) were classified as cortical regions. The frequency of each ROI, which was merely or simultaneously involved, was counted and separated according to the left and right hemispheres. For instance, a case with ASPECTS 6 actually had 4 ROI involved, therefore in theory there were four different kinds of infarction patterns as follow: 4 cortical regions, 3 cortical regions and 1 perforator region, 2 cortical regions and 2 perforator regions, and 1 cortical region and 3 perforator regions. Each of them was counted.

### Statistical Analysis

All of the clinical and imaging data, after the preliminary estimate by neurologists, radiologists, or automated-soft, would be uploaded to a pre-established, external core lab performing the final test. The inter-rater and intra-rater reliabilities of raters were graded by the value of the intraclass correlation coefficient (ICC).

Quantitative data were described as means ± SDs (*x* ± *s*) or medians and interquartile ranges (IQRs), and categorical variables were described as numbers and percentages [*n* (%)]. First, the concordance of clinical and imaging data between the right and left cerebral hemispheres was examined. Means and medians were compared using the *t*-test and the Wilcoxon rank-sum test, respectively. Categorical variables were compared using the chi-square or Fisher's exact test. As multicollinearity between subregions may affect the accuracy of the final conclusion, another test for inter-regional multicollinearity was performed, and variance inflation factor (VIF) >10 and conditional index (CI) >30 indicated a severe covariance between variables. The regions that could be associated with futile recanalization were initially assessed by a univariate model. Eventually, regions that obtained significant differences (*P* < 0.05) in the univariate model were included in a multivariate stepwise logistic regression model to identify clinical efficacy indicators for futile recanalization. Data were presented as adjusted odds ratio (OR) and 95%CI. *P* < 0.05 was considered statistically significant. All data were analyzed using SPSS 22.0 (IBM Corp, Armonk, NY, USA) and Excel (Microsoft Corp, Redmond, WI, USA) software.

## Results

### Clinical and Follow-Up Data

In total, 394 patients were screened, of whom 336 patients were finally included in the analysis with a median age of 65 (IQR, 32–78) years, and 210 (62.5%) cases were male. A total of 171 (50.89%) cases were found with at least one risk factor for atherosclerosis (diabetes, hypertension, hyperlipidemia, and smoking), combined atrial fibrillation in 167 (49.7%) cases. A total of 223 (66.36%) patients received bridging therapy, and all of them were treated with recombinant tissue plasminogen activator (rt-PA) by intravenous route. The change of NIHSS score median relative to baseline at 24 h was 7 (IQR, 4–9). Overall, 189 (56.25%) cases were in the futile recanalization group, while the other 147 (43.75%) cases were in the effective recanalization group. Besides, 52 (15.47%) cases had sICH, and type 2 of parenchymal hematoma (PH2) was identified in 34 (10.11%) cases.

**Figure 1:**
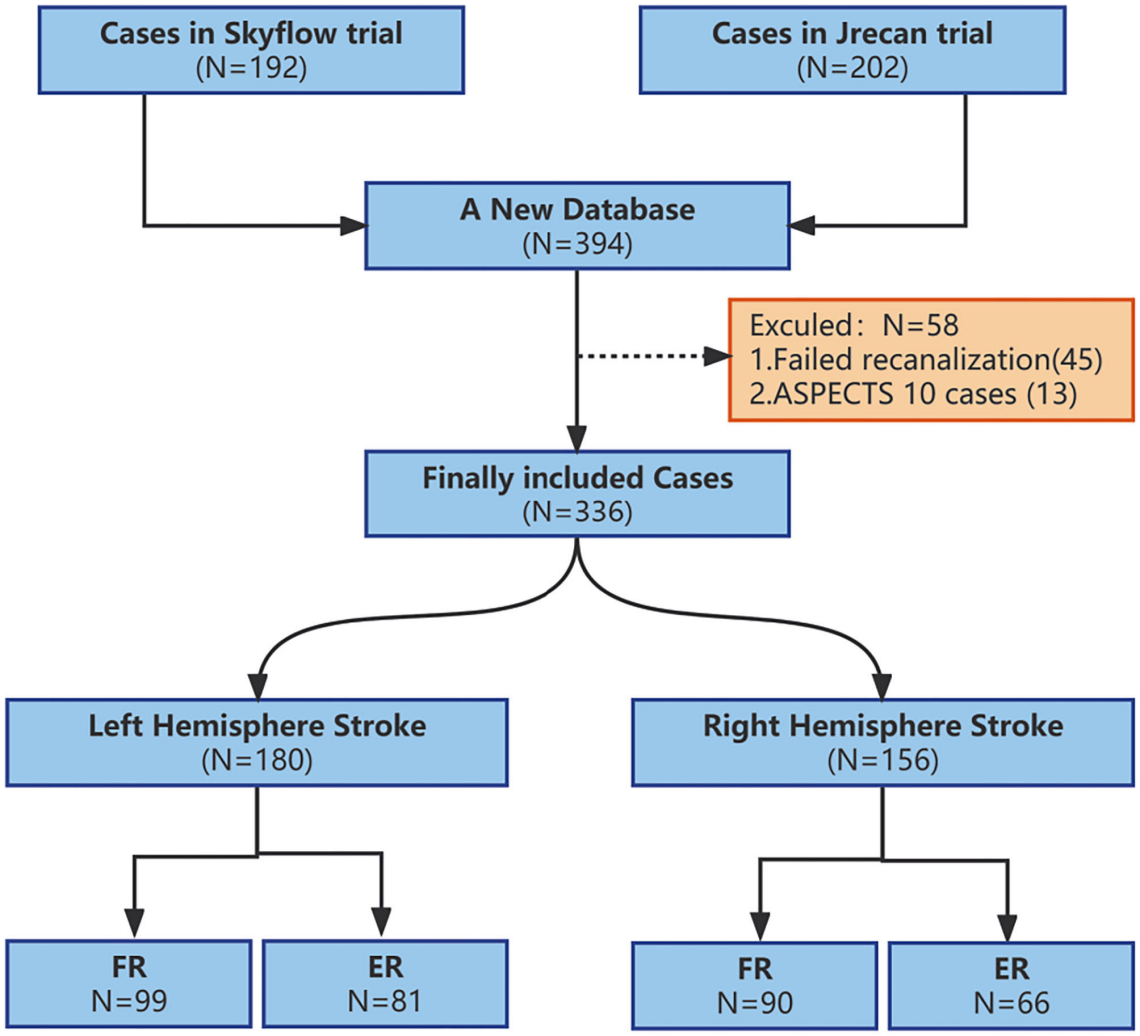
Flow chart of inclusion and exclusion at each step in the study. ASPECTS, Alberta Stroke Program Early CT score; FR, futile recanalization; ER, effective recanalization.

The stroke was located in the left hemisphere in 180 (53.57%) cases and in the right hemisphere in 156 (46.43%) cases with corresponding inclusion of 99 (55.0%) and 90 (57.68%) cases in the futile recanalization group (Figure 1). Except for in the caudate region, there was no significant difference in baseline data between the right and left cerebral hemispheres (Table 1).

**Table 1:** Comparison of the baseline data between the right and left hemispheres stroke.

**Characteristics**	**Left-hemisphere stroke (*n =* 180)**	**Right-hemisphere stroke (*n =* 156)**	***P*-values**
Age, years, median (IQR)	63(49–75)	65(50–76)	0.49
Gender (male), (*n*/%)	112(62.22%)	101(64.74%)	0.65
Risk factors for atherosclerosis, (*n*/%)	110(61.11%)	91(58.33%)	0.18
Atrial fibrillation, *n* (%)	85(47.22%)	86(55.12%)	0.23
History of antiplatelet/anticoagulant, (*n*/%)	114(63.33%)	106(67.94%)	0.05
ASPECTS, median (IQR)	8 (6–10)	8(6–9)	0.87
rt-PA, (*n*,%)	71 (39.44%)	66 (42.31%)	0.24
Infarction volume, ml, median (IQR)	23(12–31)	19(9–24)	0.07
Pretreatment NIHSS, median (IQR)	17 (12–23)	19(12–20)	0.42
Collateral circulation scale, median (IQR)	1(1–2)	1(1–2)	0.26
Time from onset to recanalization, min, median (IQR)	182 (61–301)	191(70–321)	0.11
Futile recanalization (*n*,%)	99(55.0%)	90(57.69%)	0.47
Type 2 of parenchymal hematoma, (*n*/%)	20(11.11%)	14(8.97%)	0.55
Symptomatic intracerebral hemorrhage, (*n*/%)	29(16.11%)	23(14.74%)	0.35
**Subregions of ASPECTS**
Caudate, (*n*/%)	52(28.88%)	36(23.07%)	0.04
Lenticular nucleus, (*n*/%)	34(18.88%)	30(19.23%)	0.31
Internal capsule, (*n*/%)	45(25.0%)	41(26.28%)	0.19
Insula, (*n*/%)	64(35.55%)	57(36.53%)	0.47
M1, (*n*/%)	36(20.0%)	31(19.87%)	0.25
M2, (*n*/%)	57(31.66%)	51(32.69%)	0.32
M3, (*n*/%)	31(17.22%)	31(19.87%)	0.12
M4, (*n*/%)	33(18.33%)	28(17.94%)	0.16
M5, (*n*/%)	51(28.33%)	49(31.41%)	0.32
M6, (*n*/%)	49(27.22%)	38(24.35%)	0.28

*IQR, interquartile range; ASPECTS, Alberta Stroke Program Early CT Score; NIHSS, National Institutes of Health Stroke Scale; rt-PA, recombinant tissue plasminogen activator*.

### Distribution of ASPECTS Subregions

Alberta Stroke Program Early CT score (ASPECTS) was assessed by NCCT in 197 (58.63%) cases and DWI in the remaining 139 (41.36%) cases. According to the final assessment of the external core lab, the inter-rater and intra-rater reliabilities were good for the ASPECTS location scoring. As the present study only included cases with ASPECTS ≥6, a maximum of 4 subregions were damaged in a case. Only 1 subregion was damaged in 70 cases, with the insula being the most common site (14 cases, 20.0%). Besides, 2 subregions were damaged simultaneously in 124 cases, and the combination of M5 and insula regions was the most common (33 cases, 26.61%). Moreover, 3 subregions were simultaneously involved in 92 cases, and the combination of IC, C, and I regions was the most common (16 cases, 17.39%). In addition, 4 subregions were simultaneously involved in 49 cases, with the combination of I, M2, M5, and M6 regions being the most common (9 cases, 18.23%). There were 133 cases involving both cortical and perforator areas, 119 cases involving only cortical areas, and 84 cases involving only perforator areas.

**Figure 2:**
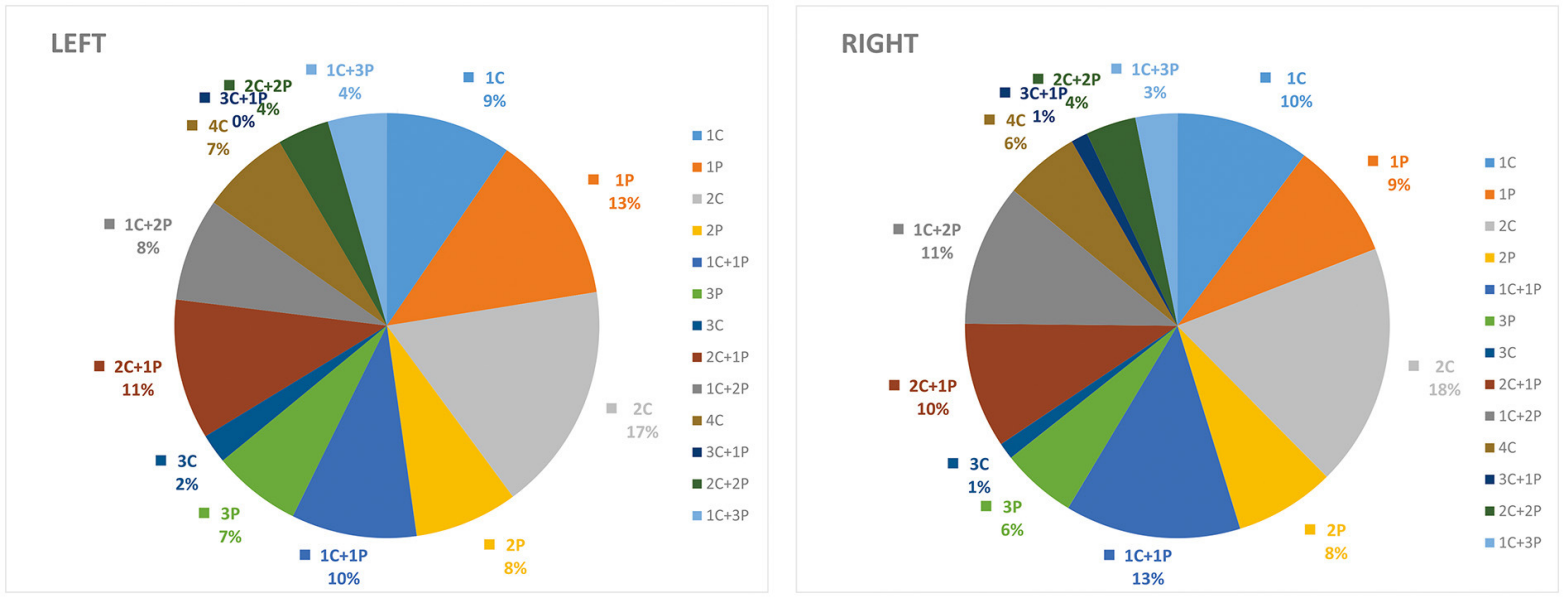
Distribution of ASPECTS subregions in the cortical (C) and deep-perforator (P) territories according to the left and right cerebral hemispheres.

The results of the statistical analysis separated by right and left hemispheres, cortical, and perforator subregions are presented in Table 2. The combined patterns of involvement in perforator and cortical subregions and their corresponding probabilities for each case were analyzed, and the results suggested that the distribution of infarct sites in perforator and cortical subregions were not significantly different according to the right and left hemispheres (Figure 2).

**Table 2:** Comparison of the distribution differences between the cortical and perforator ASPECTS subregions in the left and right cerebral hemispheres.

**Region**	**Left-hemisphere stroke**				**Right-hemisphere stroke**				***P***
***n* (%)**	**(***N =*** 180)**				**(***N =*** 156)**				
Total	C 64(35.55%)	P 49(27.22%)	C+P 67(37.22%)	–	C 55(35.25%)	P 35(22.43%)	C+P 66(42.3%)	–	0.36
1ROI	1C 17(9.44%)	1P 23(12.77%)	–	–	1C 16(10.25%)	1P 14(8.97%)	–	–	0.17
2ROI	2C 31(17.22%)	2P 14(7.77%)	1C+1P 17(9.44%)	–	2C 29(18.58%)	2P 12(7.69%)	1C+1P 21(13.46%)	–	0.24
3ROI	3C 4(2.22%)	3P 12(6.66%)	2C+1P 10(5.55%)	1C+2P 14(7.77%)	3C 2(1.28%)	3P 9(5.76%)	2C+1P 15(9.61%)	1C+2P 17(10.89%)	0.19
4ROI	4C 12(6.66%)	3C+1P 0	2C+2P 7(3.88%)	1C+3P 8(4.44%)	4C 9(5.76%)	3C+1P 2(1.28%)	2C+2P 6(3.84%)	1C+3P 5(3.21%)	0.21

*ROI, region of interest; P, perforator regions; C, cortical regions*.

### Correlation Analysis of ASPECTS Subregions and Futile Recanalization

The results of the univariate logistic regression analysis are listed in Table 3. When the analysis was performed according to the left and right hemispheres, except for the left C and right M1 subregions, the others significantly differed. Therefore, the multivariate logistic regression model was then established separately by the left and right hemispheres, and regions that obtained differences were imported into their respective models. The results showed that there were significant differences in the left IC region (OR: 1.42, 95% CI: 1.13–1.95, *P* = 0.03), the left M3 region (OR: 2.26, 95%CI: 1.36–3.52, *P* = 0.001), and the right M6 region (OR: 2.24, 95%CI: 1.32–3.36, *P* = 0.001). The goodness-of-fit results for the left-sided and right-sided models were 0.47 and 0.52, respectively (Table 3). The multicollinearity test performed before the regression analysis did not reveal the presence of collinearity between the 10 regions.

**Table 3:** Univariate and multivariate logistic regression models used to identify independent predictors of futile recanalization.

	**Left-hemisphere stroke (***N =*** 180)**				**Right-hemisphere stroke (***N =*** 156)**			
	**Univariate analysis**		**Multivariate analysis**		**Univariate analysis**		**Multivariate analysis**	
	**OR (95%CI)**	***P***	**Adjusted OR (95%CI)**	***P***	**OR (95%CI)**	***P***	**Adjusted OR (95%CI)**	***P***
C	1.64(1.05–2.37)	0.02	1.57(0.53–2.42)	0.36	1.13(0.82–1.79)	0.27	–	–
L	1.59(1.32–1.92)	0.03	1.69(0.68–2.97)	0.41	1.92(1.27–2.37)	0.02	1.87(0.59–2.31)	0.16
IC	1.79(1.45–2.41)	0.009	1.42(1.13–1.95)	0.03	1.67(1.38–2.27)	0.03	1.75(0.31–2.46)	0.21
I	2.07(1.35–2.57)	0.01	2.07(0.85–2.65)	0.25	1.94(1.23–2.97)	0.01	1.99(0.39–3.36)	0.22
M1	1.46(0.72–2.16)	0.23	–	–	1.65(1.16–2.18)	0.04	1.62(0.64–2.16)	0.34
M2	1.68(1.27–2.47)	0.01	1.77(0.79–2.21)	0.14	1.58(1.13–2.78)	0.02	2.67(0.28–4.18)	0.27
M3	2.16(1.42–4.32)	0.001	2.26(1.36–3.52)	0.001	1.89(1.32–2.81)	0.01	1.57(0.72–2.64)	0.12
M4	1.54(1.18–2.58)	0.02	1.67(0.41–2.74)	0.15	1.42(1.24–2.65)	0.02	1.64(0.54–2.25)	0.17
M5	1.78(1.39–2.79)	0.01	2.16(0.51–2.58)	0.13	2.51(1.18–3.62)	0.007	2.42(0.88–3.56)	0.07
M6	2.18(1.79–2.95)	0.007	2.27(0.78–3.29)	0.11	2.96(1.84–4.78)	0.001	2.24(1.32–3.36)	0.001

*C, indicates caudate; IC, internal capsule; L, lentiform; I, insula*.

When the analysis was carried out according to the ASPECTS image acquisition methods, the conclusions remained consistent when using the DWI. However, only the left M3 region (OR: 1.36, 95%CI: 1.21–2.26, *P* = 0.02) and the right M6 region (OR: 1.83, 95%CI: 1.13–2.74, *P* = 0.01) remained significantly different when the NCCT was used (Table 4).

**Table 4:** Multivariate logistic regression analysis used to identify independent predictors of futile recanalization stratified by the ASPECTS imaging acquisition (CT/MRI).

	**CT-based patients (***N =*** 197)**				**MRI-based patients (***N =*** 139)**			
	**Left hemisphere stroke**		**Right hemisphere stroke**		**Left hemisphere stroke**		**Right hemisphere stroke**	
	**(***N =*** 102)**		**(***N =*** 95)**		**(***N =*** 73)**		**(***N =*** 66)**	
	**Adjusted OR (95%CI)**	***P***	**Adjusted OR (95%CI)**	***P***	**Adjusted OR (95%CI)**	***P***	**Adjusted OR (95%CI)**	***P***
C	1.46(0.33–2.48)	0.27	–	–	1.55(0.57–2.49)	0.36	–	–
L	1.73(0.57–2.76)	0.38	1.92(0.45–2.67)	0.19	1.86(0.72–3.39)	0.37	1.67(0.41–2.32)	0.12
IC	1.56(0.62–1.97)	0.18	1.56(0.36–2.74)	0.22	1.53(1.21–2.57)	0.04	1.94(0.44–2.87)	0.26
I	2.14(0.76–2.57)	0.26	1.87(0.44–3.15)	0.23	1.97(0.65–2.77)	0.18	1.89(0.39–3.18)	0.17
M1	–	–	1.64(0.67–2.21)	0.35	–	–	1.68(0.54–2.08)	0.33
M2	1.73(0.72–2.34)	0.16	2.56(0.31–4.11)	0.26	1.80(0.69–2.24)	0.14	2.62(0.38–4.23)	0.28
M3	1.36(1.21–2.26)	0.02	1.66(0.65–2.67)	0.14	2.12(1.18–3.86)	0.001	1.78(0.77–2.41)	0.13
M4	1.59(0.39–2.47)	0.21	1.67(0.46–2.58)	0.19	1.74(0.44–2.87)	0.16	1.71(0.36–3.17)	0.14
M5	2.13(0.46–2.62)	0.17	2.26(0.93–3.56)	0.07	2.16(0.62–2.38)	0.22	2.42(0.82–3.73)	0.09
M6	1.98(0.74–3.25)	0.15	1.83(1.13–2.74)	0.01	2.27(0.81–3.31)	0.16	2.45(1.33–4.26)	0.001

*C, indicates caudate; IC, internal capsule; L, lentiform; I, insula*.

We also repeated the analyses using sICH as an outcome. There was no significant association between sICH and ASPECTS regions, but Left-M3, Left-L, and Right-M6 regions had trends developing sICH in the data, the corresponding *P* values were 0.06, 0.07, and 0.07 (Supplementary Table 2).

## Discussion

In the present study, we attempted to assess the efficacy of different infarct locations in predicting futile recanalization of patients with AIS due to LVO. To our knowledge, such a study design could be the first of its kind on futile recanalization with data consisting of RCT cases. The results confirmed our hypothesis that the efficacy was unequal. When the analysis was separately performed in the cerebral hemispheres, the left IC, the left M3, and the right M6 ASPECTS subregions were found as independent risk factors for the occurrence of futile recanalization after EVT if preoperative infarction had already occurred in those sites.

Previous studies mainly concentrated on the effect of infarct core volume in predicting the prognosis (1, 4, 5). In recent years, studies also suggested a close correlation between the degree of damage to the infarct core and its distribution and prognosis. Rosso et al. demonstrated that infarct sites had even a greater influence on prognosis than infarct volume (21). BYQ Tan reported that the M5 region served as a reliable biomarker for predicting prognosis in thrombolyzed patients with AIS (22). However, there were few relevant studies focusing on EVT, and although they mainly used the ASPECTS to mark the infarct sites, their results were inconsistent (7, 21). The reasons may be that these studies were mostly retrospective case-control studies with small sample sizes, enrollment of cases was time-consuming, and outdated or inconsistent devices and techniques were used (5, 7, 19, 21). The data in the present study were obtained from two large sample sizes, multicenter, prospective, and RCTs with a strict trial design and rigorous protocol performed using the mainstream EVT devices and techniques, with a unified and standardized evaluation and supervision system, ensuring the accuracy and homogeneity of the data. Another important reason may be the inconsistency of imaging assessment methods, in which some studies used manual measurement of NCCT-ASPECTS. Related studies have compared the accuracy of NCCT-ASPECTS and DWI-ASPECTS, *via* manual measurement and automated (software-dependent) measurement, suggesting that manual measurement of NCCT-ASPECTS had worse accuracy compared with automated measurement (17, 18). Therefore, all NCCT-ASPECTS in the present study were measured automatically by software, to minimize the measurement errors.

The infarct distribution in cortical and perforator areas did not significantly differ between the left and right cerebral hemispheres in the present study, which was inconsistent with previous reports. Charlotte et al. suggested that the perforator regions were more rarely involved in the right hemisphere compared with that in the left hemisphere (21). This could be related to the fact that the diameter of the right common carotid artery was thicker and closer to the ascending aorta than the left one, thus, cardioembolism was more likely to affect the cortical area in the right hemisphere. However, cases in the present study were all from Asia who had a significantly higher incidence of intracranial large artery atherosclerosis compared with European and American populations (23), and this difference may contribute to the different distributions of infarct sites.

The present study also found significant differences in the imaging results, the IC subregion did not emerge as a stable predictor as expected, and this phenomenon has been previously reported (24). Some reasons could explain it as follows. (1) The posterior limb of the IC is mainly damaged when the terminal segment of the internal carotid artery is occluded and the anterior choroidal artery is involved, while the overall incidence of this subtype is lower, thus, that it may reduce its performance for predicting the overall prognosis. (2) All patients in the current study were in the early time window, and as the measurement sensitivity of DWI was better than NCCT, the false-negative rate was higher in the NCCT group compared with the DWI group. In contrast, the M6 and M3 areas were very stable in predicting poor outcomes, this was consistent with the pooled analysis, which reported the M6 subregion was the strongest predictor in almost all of the subgroup analyses (25).

The pathophysiological basis for the correlation between a particular site of ASPECTS and prognosis is still the regional distribution of brain functions. For instance, the IC is classically described as a structure that contains motor fibers from different brain regions, M6 and M5 areas are adjacent to the cortical motor function areas, and an infarct can cause permanent motor function impairment. Meanwhile, damage to the right M6 area can cause symptoms of neglect, which can also affect motor function (26). The left M3 area is closely associated with spatial resolution, attention, etc. There is a strong correlation between the insula and emotional experience (27, 28). Meanwhile, some regions did not show significant differences in the current study, but this does not mean they have no correlations with the prognosis. One of the possible reasons is that although the mRS score is generally used as an indicator to assess outcomes, its evaluation system is relatively crude and is more significantly influenced by some functions, such as movement and attention.

Our study has some limitations. First, although patients were prospectively registered, all data of this analysis were assessed retrospectively. Second, patients in the late window, which have a noticeable proportion in the real world, were not included. However, given the time of symptom onset to recanalization is an important factor affecting outcome and patients in the early window suffer from a shorter recanalization time, this may reduce the influence of the time factor. Third, the ASPECTS subregions only cover most of the blood supply areas of the middle cerebral artery, while some other areas, such as the centrum semiovale and white matter region, are not involved in the ASPECTS system, which are also correlated with prognosis. Fifth, early DWI/NCCT lesion reversal is not a rare phenomenon after EVT, whether it is transient or sustained (29). This is a challenge to the classical definition of infarct core and also affects the assessment of the prognosis. Sixth, in addition to the infarct location, there are many other factors that can influence futile recanalization, such as infarct growth. The lack of assessment of these factors and their reactions to ASPECTS regions may result in an overestimation of the infarct location effect (30). Seventh, some items of the study design may result in bias, for instance, excluding ASPECTS 10 patients and rating ASPECTS with different methods. In the future, RCTs with larger sample sizes and a more effective prediction system will be required to guide clinical decisions and reduce the incidence of futile recanalization.

## Conclusions

We demonstrate that the efficacy of different infarct locations in predicting futile recanalization is different in patients with AIS. When the analysis was separately performed in the cerebral hemispheres, the left IC, the left M3, and the right M6 ASPECTS subregions were found as independent risk factors for the occurrence of futile recanalization. And these regions preoperatively in the infarction core or penumbra cites may be helpful for guiding the endovascular thrombectomy decision-making. Further studies are necessary to validate the prognostic value of this finding in combination with other clinical factors for futile recanalization.

## Data Availability Statement

The original contributions presented in the study are included in the article/Supplementary Material, further inquiries can be directed to the corresponding author/s.

## Ethics Statement

The studies involving human participants were reviewed and approved by Ethics Committee of Drugs (Devices) Clinical Experiment of Henan Provincial People's Hospital, Nanyang Central Hospital, The First Affiliated Hospital of Xi'an Jiaotong University. The patients/participants provided their written informed consent to participate in this study. Written informed consent was obtained from the individual(s) for the publication of any potentially identifiable images or data included in this article.

## Author Contributions

Z-sL and T-xL contributed to study conception and design. Z-sL, T-fZ, Y-kH, C-mW, J-fH, and QL contributed to acquisition, data interpretation, and analysis. Z-sL and H-lZ drafted the manuscript. Z-lW and L-fZ contributed significant intellectual content. All authors made a significant contribution to the study and manuscript preparation, critically revised the paper, and approved the final version of the manuscript.

## Funding

This trial was supported by Provincial and Ministerial Joint Important Project of Provincial Medical Science and Technology (SBGJ202102036) and Provincial and Ministerial Joint Important Project of Provincial Medical Science and Technology (SBGJ202002001).

## Conflict of Interest

The authors declare that the research was conducted in the absence of any commercial or financial relationships that could be construed as a potential conflict of interest.

## Publisher's Note

All claims expressed in this article are solely those of the authors and do not necessarily represent those of their affiliated organizations, or those of the publisher, the editors and the reviewers. Any product that may be evaluated in this article, or claim that may be made by its manufacturer, is not guaranteed or endorsed by the publisher.
