# Supplementary material for: Unequal Efficacy of Different Infarct Location in Predicting Futile Recanalization of Patients With Acute Ischemic Stroke
Source: Front Neurol. 2022 Aug 26;13:928773. doi: 10.3389/fneur.2022.928773 (PMC9462394; doi:10.3389/fneur.2022.928773)
Supplement: Supplementary file 1 [file Table_1.DOCX]

Supplemental Table 1:The list of recruitment centers of both trails.

|  | Sequence number | Centers |
| --- | --- | --- |
| Skyflow study | 1 | Henan Provincial People's Hospital |
|  | 2 | Nanjing First Hospital |
|  | 3 | Shanghai Tongji Hospital |
|  | 4 | The Second Affiliated Hospital of Zhejiang University of Medicine |
|  | 5 | The First Affiliated Hospital of Nanjing Medical Medical University |
|  | 6 | Baotou Central Hospital |
|  | 7 | The Affiliated Hospital of Nantong University |
|  | 8 | Hunan Provincial People's Hospital |
|  | 9 | The First Affiliated Hospital of Soochow University |
|  | 10 | The Brain Hospital of Hunan Province |
|  | 11 | Zhengzhou Central Hospital |
|  | 12 | Nanning Second People's Hospital |
|  | 13 | Nanyang Central Hospital |
| Jrecan study | 1 | Henan Provincial People's Hospital |
|  | 2 | The Third Affiliatd Hospital of Guangzhou Medical Hospital |
|  | 3 | The First Affiliated Hospital of Henan University of Science and Technology |
|  | 4 | The First Affiliated Hospital of Jinan University |
|  | 5 | Luoyang Central Hospital |
|  | 6 | Nanjing First Hospital |
|  | 7 | Puyang Oilfield General Hospital |
|  | 8 | Shunde Hospital of Southern Medical University |
|  | 9 | Lishui Central Hospital |
|  | 10 | The First Affiliated Hospital of Xi'an Jiaotong University |
|  | 11 | The Second Affiliatd Hospital of Guangzhou Medical Hospital |
|  | 12 | Nanyang Central Hospital |
|  | 13 | The First Hospital of Jilin University |
|  | 14 | The China-Japan Union Hospital of Jilin University |

Supplemental Table 2: Univariate models used to analyze differences in each ASPECTS sub-region affecting symptomatic intracerebral hemorrhage.

|  | **Left-hemisphere Stroke**  **(N=180)** | | |  | **Right-hemisphere stroke**  **(N=156)** | | |
| --- | --- | --- | --- | --- | --- | --- | --- |
| **Region** | **sICH**  **(N=29)** | **No-sICH**  **(N=151)** | **P-values** | **Region** | **sICH**  **(N=23)** | **No-sICH**  **(N=133)** | **P-values** |
| C | 11(37.93%) | 41(27.15%) | 0.24 | C | 8(34.78%) | 28(21.05%) | 0.15 |
| L | 9(56.57%) | 25(49.38%) | 0.07 | L | 7(30.43%) | 23(17.29%) | 0.16 |
| IC | 8(27.58%) | 37(24.51%) | 0.73 | IC | 4(17.39%) | 37(27.82%) | 0.29 |
| I | 14(48.27%) | 50(33.11%) | 0.12 | I | 11(47.83%) | 46(34.59%) | 0.22 |
| M1 | 9(56.57%) | 27(17.88%) | 0.11 | M1 | 6(26.9%) | 25(18.80%) | 0.41 |
| M2 | 13(44.82%) | 44(29.14%) | 0.09 | M2 | 9(39.13%) | 39(29.32%) | 0.35 |
| M3 | 9(31.03%) | 22(14.57%) | 0.06 | M3 | 8(34.78%) | 23(17.29%) | 0.08 |
| M4 | 7(24.13%) | 26(17.22%) | 0.33 | M4 | 7(30.43%) | 21(15.79%) | 0.14 |
| M5 | 12(41.38%) | 39(25.83%) | 0.09 | M5 | 4(17.39%) | 45(33.83%) | 0.17 |
| M6 | 10(34.48%) | 39(25.83%) | 0.34 | M6 | 9(39.13%) | 29(21.80%) | 0.07 |
| C, indicates caudate; IC, internal capsule; L, lentiform; I, insula; sICH, symptomatic intracerebral hemorrhage. | | | | | | | |
