# Supplementary material for: Unequal Efficacy of Different Infarct Location in Predicting Futile Recanalization of Patients With Acute Ischemic Stroke
Source: Front Neurol. 2022 Aug 26;13:928773. doi: 10.3389/fneur.2022.928773 (PMC9462394; doi:10.3389/fneur.2022.928773)
Supplement: Supplementary file 2 [file Presentation_1.pdf]

编号：AF/SC-07/03.1

伦理审查意见

意见号	2017-034-01		
项目名称	取栓装置用于急性缺血性卒中血管内治疗的有效性及安全性研究		
项目来源	心凯诺医疗科技(上海）有限公司		
研究单位	河南省人民医院		
主要研究者	李天晓		
审查类别	初始审查	审查方式	会议审查
审查日期	2017.10.19	审查地点	科教大厦25楼小会议室
审查委员	邵凤民、刘广芝、王宇明、孟凡民、杨玉秀、王丽霞、赵丽敏、张 伟、高尚刚、李 磊		
审查文件	1. 伦理审查申请接洽函<br>2. 初始审查申请<br>3. 临床试验方案（版本号：1.0　版本日期：2017年8月25日）<br>4. 知情同意书（版本号：1.1　版本日期：2017年10月10日）<br>5. 研究病历和病例报告表<br>5.1 研究病历（版本号：1.0　版本日期：2017年8月25日）<br>5.2 病例报告表（版本号：1.0　版本日期：2017年8月25日）<br>6. 研究者手册<br>7. 产品说明书<br>7.1 取栓装置说明书<br>7.2 血流重建装置说明书<br>8. 自检报告和产品注册检测报告<br>8.1 自检报告<br>8.2 产品注册检测报告<br>9. 取栓装置产品技术要求<br>10. 主要研究者资质证明文件<br>11. 临床试验团队成员表、团队成员资质证明文件<br>12. 临床试验专业的设施和条件能满足试验的综述<br>13. 试验用医疗器械的研制符合适用的医疗器械质量管理体系相关要求的声明<br>14. 医疗器械动物实验报告<br>15. 保险合同<br>16. 申办者资质证明		

	17. 申办者对监查员及项目经理的授权书及 GCP 证书
**审查决定**	作必要的修正后同意

审查意见

根据卫计委《涉及人的生物医学研究伦理审查办法》(2016)、CFDA《药物临床试验质量管理规范（2003）》、《医疗器械临床试验质量管理规范（2016）》、《体外诊断试剂临床试验技术指导原则（2014）》、WMA《赫尔辛基宣言》和 CIOMS《人体生物医学研究国际道德指南》的伦理原则，经本伦理委员会审查，意见如下：

作必要的修正后同意。

按审查意见修改后的文件，或对审查意见不同观点的陈诉，请提交“复审申请”，方案/知情同意书请注明新的版本号和版本日期，并以阴影和/或下划线方式标注修改部分，报伦理委员会审查，经批准后执行。

**调整的年度/定期跟踪审查频率**	12 个月
**伦理委员会**	河南省人民医院药物（器械）临床试验伦理委员会
**主任委员签字**	[signature]
**日期**	2017.10.23.

编号：AF/SC-08/03.1

河南省人民医院药物（器械）临床试验伦理委员会
伦理审查批件

批件号	2017-034-02		
项目名称	取栓装置用于急性缺血性卒中血管内治疗的有效性及安全性研究		
项目来源	心凯诺医疗科技（上海）有限公司		
试验产品名称/类别	取栓装置/III类		
研究单位	河南省人民医院	承担科室	介入科
主要研究者	李天晓	职称	主任医师
审查类别	审查方式	审查日期	
初始审查	会议审查	2017.10.19	
复审	快速审查	2017.11.08	
审查地点	科教大厦 26 楼、投诉办		
审查委员	王宇明、李磊		
审查文件	初始审查<br>1. 伦理审查申请接洽函<br>2. 初始审查申请<br>3. 临床试验方案（版本号：1.0　版本日期：2017 年 8 月 25 日）<br>4. 知情同意书（版本号：1.1　版本日期：2017 年 10 月 10 日）<br>5. 研究病历和病例报告表<br>5.1 研究病历（版本号：1.0　版本日期：2017 年 8 月 25 日）<br>5.2 病例报告表（版本号：1.0　版本日期：2017 年 8 月 25 日）<br>6. 研究者手册<br>7. 产品说明书<br>7.1 取栓装置说明书<br>7.2 血流重建装置说明书<br>8. 自检报告和产品注册检测报告<br>8.1 自检报告<br>8.2 产品注册检测报告<br>9. 取栓装置产品技术要求<br>10. 主要研究者资质证明文件<br>11. 临床试验团队成员表、团队成员资质证明文件<br>12. 临床试验专业的设施和条件能满足试验的综述<br>13. 试验用医疗器械的研制符合适用的医疗器械质量管理体系相关要求的声明<br>14. 医疗器械动物实验报告<br>15. 保险合同<br>16. 申办者资质证明<br>17. 申办者对监查员及项目经理的授权书及 GCP 证书<br>复审<br>1.复审申请<br>2.知情同意书（版本号：1.2　版本日期 2017 年 10 月 25 日）		

	3.知情同意书修改说明函

审查意见：同意

根据卫计委《涉及人的生物医学研究伦理审查办法》（2016）、CFDA《药物临床试验质量管理规范（2003）》、《医疗器械临床试验质量管理规范（2016）》、《体外诊断试剂临床试验技术指导原则（2014）》、WMA《赫尔辛基宣言》和CIOMS《人体生物医学研究国际道德指南》的伦理原则，经本伦理委员会审查，同意按所批准的临床研究方案、知情同意书、招募材料开展本项研究。

请遵循GCP原则、遵循伦理委员会批准的方案开展临床研究，保护受试者的健康与权利。

研究开始前，请申请人完成临床试验注册。

研究过程中若变更主要研究者，对临床研究方案、知情同意书、招募材料等的任何修改，请申请人提交修正案审查申请。

发生严重不良事件，请申请人及时提交严重不良事件报告。

请按照伦理委员会规定的年度/定期跟踪审查频率，申请人在截止日期前1个月提交研究进展报告；申办者应当向组长单位伦理委员会提交各中心研究进展的汇总报告；当出现任何可能显著影响试验进行、或增加受试者危险的情况时，请申请人及时向伦理委员会提交书面报告。

研究纳入了不符合纳入标准或符合排除标准的受试者，符合中止试验规定而未让受试者退出研究，给予错误治疗或剂量，给予方案禁止的合并用药等没有遵从方案开展研究的情况；或可能对受试者的权益/健康、以及研究的科学性造成不良影响等违背GCP原则的情况，请申办者/监查员/研究者提交违背方案报告。

申请人暂停或提前终止临床研究，请及时提交暂停/终止研究报告。

完成临床研究，请申请人提交研究完成报告。

本项临床试验应当在批准之日起一年内实施，逾期未实施的，本批件自行废止。

年度/定期跟踪审查频率	12个月
有效期	1年；截止日2018年11月08日<br>（如试验逾期未实施，需再次提交初始审查申请）
通讯地址<br>联系人与联系电话	河南省郑州市纬五路7号　450003<br>陈明月　0371-87160817
主任委员签字	（签名）
伦理委员会	河南省人民医院药物（器械）临床试验伦理委员会（盖章）
日期	2017.11.13.

南阳市中心医院伦理委员会

医疗器械临床试验伦理审查批件

批件号：2018-002-01

<table>
<tr><td>项目名称</td><td colspan="4">取栓装置用于急性缺血性卒中血管内治疗的有效性及安全性研究</td></tr>
<tr><td>器械名称</td><td colspan="2">取栓装置</td><td>器械型号</td><td>TD-320 TD-420 TD-430
TD-440 TD-620 TD-630</td></tr>
<tr><td>申办单位</td><td colspan="2">心凯诺医疗科技（上海）有限公司</td><td>评价目的</td><td>评价取栓装置的安全性及有效性</td></tr>
<tr><td rowspan="2">临床申请研究单位（基地专业）</td><td rowspan="2">神经内科</td><td colspan="2">专业负责人</td><td>温昌明</td></tr>
<tr><td colspan="2">项目负责人</td><td>温昌明</td></tr>
<tr><td rowspan="18">报送材料</td><td colspan="4">伦理审查申请表</td></tr>
<tr><td colspan="4">取栓装置注册检验报告</td></tr>
<tr><td colspan="4">取栓装置产品技术要求</td></tr>
<tr><td colspan="4">试验用医疗器械／对照用医疗器械说明书</td></tr>
<tr><td colspan="4">临床试验方案（1.1，2018 年 9 月 20 日）</td></tr>
<tr><td colspan="4">研究者手册（1.0，2017 年 8 月 25 日）</td></tr>
<tr><td colspan="4">病例报告表（1.0，2017 年 8 月 25 日）</td></tr>
<tr><td colspan="4">EDC 空白模版</td></tr>
<tr><td colspan="4">研究病历（1.1，2018 年 9 月 20 日）</td></tr>
<tr><td colspan="4">知情同意书（1.3，2018 年 11 月 8 日）</td></tr>
<tr><td colspan="4">研究者资格证明文件／研究小组名单</td></tr>
<tr><td colspan="4">临床试验机构的设施条件能够满足试验要求的综述</td></tr>
<tr><td colspan="4">产品质检报告</td></tr>
<tr><td colspan="4">医疗器械临床试验委托书</td></tr>
<tr><td colspan="4">申办者营业执照／关于无医疗器械生产许可证的说明</td></tr>
<tr><td colspan="4">试验用医疗器械的研制符合适用的医疗器械质量管理体系相关要求的说明</td></tr>
<tr><td colspan="4">申办者保证所提供资料真实性的声明</td></tr>
<tr><td colspan="4">研究者保证所提供资料真实性的声明</td></tr>
</table>

<table>
<tr><td rowspan="3"></td><td colspan="5">取栓装置动物实验报告</td></tr>
<tr><td colspan="5">组长单位伦理审查批件</td></tr>
<tr><td colspan="5">临床试验保险保单摘要</td></tr>
<tr><td colspan="6">*依据 GCP 要求及相关法规，本伦理委员会的组织和实施相对独立
*本院伦理委员会的人员组成和工作程序是符合 GCP 原则以及国家相关规定的</td></tr>
<tr><td colspan="2">伦理委员会出席人数（ 8 ）人</td><td colspan="2">回避人数（ 0 ）人</td><td colspan="2">弃权人数（ 0 ）人</td></tr>
<tr><td rowspan="2">结论</td><td>同意 8 票</td><td colspan="2">不同意 0 票</td><td colspan="2">作必要修改后同意 0 票</td></tr>
<tr><td colspan="2">作必要修改后重审 0 票</td><td colspan="2">终止或暂停试验 0 票</td><td>回避 0 票</td></tr>
<tr><td colspan="6">审批意见：同意

主任委员：[signature]
2018 年 11 月 23 日</td></tr>
</table>

注：1、批件有效期 1 年（截止 2019 年 11 月 22 日），跟踪审查频率 12 个月

2、联系方式：南阳市卧龙区工农路 312 号，0377-63200149

会议签到表

Sign-in Sheet of Full IRB Meeting Templates

地点：南阳市中心医院三号楼三楼第一会议室

日期：2018 年 11 月 23 日

姓名	性别	职务	专业情况	签 名
李玉东	男	主任委员	心血管内科 主任医师	请假
张保朝	男	副主任委员	神经内科 主任医师	[illegible]
付莉萍	女	委员	医务科 主任	[illegible]
于桂青	女	委员	护理部 主任护师	[illegible]
门永忠	男	委员	超声诊断科 主任医师	[illegible]
曾宪强	男	委员	放射科 主任医师	[illegible]
雷典	男	委员	律师	雷典
郑芝欣	女	委员	药学部 主任药师	请假
王霞	女	委员	护理部 主任护师	王霞
杨如玉	女	委员	血液内科 主任医师	请假
杨侃	男	委员	心血管外科 主任医师	请假
郑喜胜	男	委员	重症医学科 副主任医师	[illegible]

伦理委员会审查批件

受理号： YWLLSL-2018-059-08　　　　批件号： 2018 伦审药临字第（82）号-1

No： XJTU1AF2018LSY-82-1

项目名称及方案号	取栓装置用于急性缺血性卒中血管内治疗的有效性及安全性研究/ TD-LC-001				
申办者	心凯诺医疗科技（上海）有限公司				
型检报告号	国医检(械)字 ZC2017 第 159 号(TD-420) 国医检(械)字 ZC2017 第 269 号(TD-320) 国医检(械)字 ZC2017 第 272 号(TD-630)		器械名称	取栓装置	
器械分类	II类□　III类■　体外诊断试剂□				
承担科室	神经内科	主要研究者	韩建峰	职称	副主任医师
审查时间	2018-12-20		审查地点	行政三楼会议室	
审查类别	会议审查■　快速审查□　初始审查□　跟踪审查■　复审□				
送审文件	见附件				
审查委员	见委员签到表				

审查意见	同意	作必要修正后同意	作必要修正后重审	不同意	终止或暂停
	■	□	□	□	□
	该研究进行过程中，伦理委员会进行定期跟踪审查，审查频率：<br>3 个月 □　6 个月 □　9 个月 □　12 个月 ■<br>主任委员签名：　　　　日期：2018-12-26				

注意事项：1.本研究应当在伦理委员会批准之日起 1 年内实施，逾期未实施的，本批件自行作废。

2. 已批准项目须遵循本伦理委员会批准的方案执行，需符合中国 GCP、ICH-GCP 和《赫尔辛基宣言》原则。

3 该临床试验项目如涉及人类遗传资源国际合作或者按照相关规定必须经有关部门专项审批的内容，需获得相应部门审批后方可开展项目，其批件应递交伦理备案。

4.本伦理委员会仅对中文材料审查，申办方对中文翻译件负责，如有出入，以中文为准。

5 研究过程中,对已批准的临床研究方案、知情同意书、招募材料及其他提供给受试者的资料等任何修或主要研究者更换等。请及时递交“修正案审查”申请，获得批准后方可执行。

6 根据本伦理委员会的定期跟踪审查频率，请在审查日到期前一个月提交“定期跟踪审查报告”。

7.暂停/提前终止临床研究，请及时向伦理委员会提交报告。

8. 发生严重不良事件及影响研究风险受益比的非预期事件，请及时报告本伦理委员会。

9. 发生重大方案违背情况须及时报告本伦理委员会。

10. 完成临床研究，请提交结题报告。

本伦理委员会严格遵循 ICH-GCP、GCP 和相关法规的要求进行构建、运作、实施各项操作程序。联系地址：西安市雁塔西路 277 号联系人：张彩霞　电话/传真： 029- 85323473

附件

序号	文件名	版本号/日期
1.	组长单位修正案审查意见	NA
2.	研究病历	1.1/2018-09-20
3.	临床试验方案	1.1/2018-09-20
4.	试验方案修订说明	NA
5.	知情同意书修订说明	NA
6.	研究病历修订说明	NA
7.	知情同意书	西安交通大学第一附属医院专用版 1.1/2018-11-08

西安交通大学第一附属医院医学伦理委员会

伦理审查会议参会委员名单

地点：行政三楼会议室　　　　　　　　　　日期：2018 年 12 月 20 日

姓　名	职务	性别	工作单位	专　业	职　称	签　名
杨　岚	主任委员	女	一附院	呼吸内科	教授/主任医师	
董亚琳	副主任委员	女	一附院	临床药理	教授/主任药师	
尹爱萍	委员	女	一附院	肾内科	主任医师	
邱裕生	委员	男	一附院	骨科	研究员/主任医师	
郑雪梅	委员	女	一附院	护理部	主任护理师	
王明旭	委员	男	医学院	伦理学	教　授	
宋　丽	委员	女	永嘉信律师事务所	法学	律　师	
陈丽梅	委员	女	一附院	血液科	主任医师	
高成阁	委员	女	一附院	精神科	主任医师	
杨　谨	委员	女	一附院	肿瘤学	教授/主任医师	
白　玲	委员	女	一附院	心内科	主任医师	
王　陵	委员	女	第四军医大学	卫生统计学	副教授	
李　健	委员	男	陕师大		居　民	
